# Influence of *Agaricus bisporus* establishment and fungicidal treatments on casing soil metataxonomy during mushroom cultivation

**DOI:** 10.1186/s12864-022-08638-x

**Published:** 2022-06-15

**Authors:** Maria Luisa Tello Martín, Rebeca Lavega, Jaime Carrasco Carrasco, Margarita Pérez, Antonio J. Pérez-Pulido, Michael Thon, Ernesto Pérez Benito

**Affiliations:** 1 Mushroom Technological Research Center of La Rioja (CTICH), Ctra. Calahorra km 4, 26560 Autol, La Rioja Spain; 2 Department of Plant Sciences, University of Oxford, SParks Rd, Oxford, OX1 3RB UK; 3 Andalusian Centre for Developmental Biology (CABD, UPO-CSIC-JA). Faculty of Experimental Sciences (Genetics Dept.), University Pablo de Olavide (Sevilla), 41013 Sevilla, Spain; 4 Universidad de Salamanca, Instituto de Investigación en Agrobiotecnología (CIALE), Calle Río Duero 12, 37185 Villamayor, Salamanca Spain

**Keywords:** Mushroom, Metataxonomy, Microbiota, *Agaricus*, QIIME2, 16S, ITS

## Abstract

The cultivation of edible mushroom is an emerging sector with a potential yet to be discovered. Unlike plants, it is a less developed agriculture where many studies are lacking to optimize the cultivation. In this work we have employed high-throughput techniques by next generation sequencing to screen the microbial structure of casing soil employed in mushroom cultivation (*Agaricus bisporus*) while sequencing V3-V4 of the 16S rRNA gene for bacteria and the ITS2 region of rRNA for. In addition, the microbiota dynamics and evolution (bacterial and fungal communities) in peat-based casing along the process of incubation of *A. bisporus* have been studied, while comparing the effect of fungicide treatment (chlorothalonil and metrafenone). Statistically significant changes in populations of bacteria and fungi were observed. Microbial composition differed significantly based on incubation day, changing radically from the original communities in the raw material to a specific microbial composition driven by the *A. bisporus* mycelium growth. Chlorothalonil treatment seems to delay casing colonization by *A. bisporus*. *Proteobacteria* and *Bacteroidota* appeared as the most dominant bacterial phyla. We observed a great change in the structure of the bacteria populations between day 0 and the following days. Fungi populations changed more gradually, with *A. bisporus* displacing the rest of the species as the cultivation cycle progresses. A better understanding of the microbial communities in the casing will hopefully allow us to increase the biological efficiency of the crop.

**Supplementary Information:**

The online version contains supplementary material available at 10.1186/s12864-022-08638-x.

## Background

Mushrooms have been cultivated since ancient times especially in the eastern countries [1]. They have been appreciated not just for their flavour but for their nutritional and medicinal value. About 7000 species of mushrooms are believed to have varying degrees of edibility, and more than 3000 species of 231 different genera are considered to be major edible mushrooms [2]. Despite this, only about 60 edible mushrooms are grown commercially, and around 10 are produced on an industrial scale. *Agaricus bisporus* (J. E. Lange) Imbach is cultivated all over the world to produce button mushrooms. These carpophores have relative high protein content and contain fiber, vitamins, minerals and bioactive compounds. *A. bisporus* is grown in compost consisting of wheat straw, horse or chicken manure [3]. After the compost is colonized, it is covered with a casing soil, which is required for maintaining moisture, developing a specific microbial flora and trapping several metabolites [4–6]. This casing soil is prepared with black or blonde peat and calcium carbonate, to correct pH 7–8. Peat is a fossil organic material, coming from deposits of vegetal organic matter (moss) accumulated over thousands of years. The use of peat in *A. bisporus* cultivation is essential, since until now no other material is known to have its characteristics: high water-holding capacity but water that is available for mushroom use, heavy structure but porous to facilitate gas exchange with a specific microbiota [7]. Peat used for mushroom growing accounts for only a small portion of the total volumes of peat extracted worldwide. However, peat is an unsustainably sourced non-renewable resource whose availability is diminishing due to reserve depletion and regulatory restrictions. Many attempts have been made to replace peat by other materials such as pine bark or coconut fibre. However, due to the particular physico-chemical properties and native microbiota, peat is still seen as the only optimal option [8]. Casing soil characteristics may also affect the development of mushroom diseases, such as dry bubble caused by *Lecanicillium fungicola* (Preuss), Zare and Gams, and wet bubble caused by *Mycogone perniciosa* (Magnus) Delacroix [9, 10]. To prevent disease occurrence, chemical products are applied. Notably, the approved fungicides for mushroom use were not specifically developed for mushroom pathogens, and therefore the mushroom industry face side problems such as reduced specificity, resistance outbreaks among pathogens and regulatory limitations for their use. Currently the number of permitted active ingredients with fungicidal action is very limited, therefore it is important to investigate in this line to find alternatives in the fight against these pathogens.

To improve mushroom cultivation methods, the role of microbial communities in the casing soil must be understood. The knowledge generated may contribute to the development of alternative materials of non-fossil origin, more environmentally friendly and with inhibiting properties of other pathogenic organisms, thus improving both the cultivated mushroom sector and other agricultural sectors that use peat, as nurseries or gardens.

In this work we have employed high-throughput techniques by next generation sequencing (NGS) to screen the microbiota dynamics and evolution (bacterial and fungal communities) in peat-based casing soil along the process of fructification of *A. bisporus*, and the impact of fungicide treatment (chlorothalonil and metrafenone) on the microbial communities. To reach these objectives two highly conserved genetic regions were sequenced in an Illumina MiSeq: V3-V4 of the 16S rRNA gene for bacteria and the ITS2 region of rRNA for fungi.

## Results

Data from the trial were divided in three data-set. The first one (data-set DAYS) represents the differences between incubation days in the control without fungicides application: casing day (D00), fungicides application (D04), ruffling day (D07) and beginning of first flush (D16). Ruffling is an agronomic technique used in mushroom farming that consists of raking the surface of the casing soil in order to aerate it and mix compost with the casing so that mushroom colonization is faster and more homogeneous. The second (data-set TREATMENTS D07) represents the differences between the control and the two fungicide treatments (chlorothalonil and metrafenone) at ruffling day (D07). And finally, the third (data-set TREATMENTS D16) represents the differences between the control and the two fungicide treatments in the beginning of the first flush (D16). For these three data-set both bacterial and fungi communities have been analysed.

### Sequencing and assembly

#### Bacterial 16S analysis

For both, the Bacterial 16S analysis and the Fungi ITS2 analysis, all samples (*n* = 24) were sequenced on the Illumina MiSeq. Sample 26 (G9) from the Bacterial 16S analysis, was excluded from the analysis because of the low number of reads obtained (1168 reads). All data from the metataxonomic analysis are collected in Table 1.

**Table 1:** Metataxonomic analysis data

Analysis	Bacterial 16S	Fungi ITS2
Number of samples	23	24
Total read pairs	3,935,266	8,407,044
Total read pairs average	178,875	350,293
Total read pairs SD (+/−)	68,328	262,317
Truncation selected (forward/reverse)	260/230	280/200
ASV’s classified after DADA2	14,018	2533
ASV’s classified after filtering	3074	950

Alpha rarefaction curves were analysed (Supplementary material Figs. 7 and 8), to confirm that sampling depth was enough to observe the full community diversity. Alpha and beta diversity box-and-whisker plots and statistics are collected in Supplementary material Figs. 1, 2, 3, 4, 5 and 6.

#### Analysis data-set DAYS

First, we performed a metataxomonic analysis to study the different bacterial populations. Phylum and family level were visualized in barplots. Then, we analysed the diversity to check if there were significant differences within samples (alpha diversity) and between samples (beta diversity). Finally, we carried out an ANCOM test to identify different abundances across bacterial samples at genus level.

**Fig. 1:**
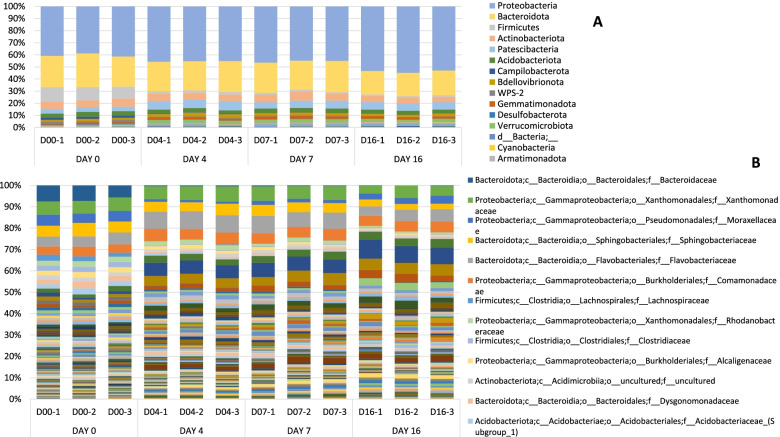
Barplot with the Bacterial 16S metataxonomical classification. Legend: Bacterial 16S metataxonomical classification by days at Phylum level (**A**) and at Family level (**B**)

The most highly represented bacteria phylum was *Proteobacteria* and *Bacteroidota* for all samples in data-set DAYS (Fig. 1A). *Proteobacteria* at D00 appears at 40.34%+/− 1.39 SD (percentage of ASVs classified) and increases along days, with a percentage of 53.70%+/− 0.96 SD at D16. *Firminutes* phylum presents a 10.88%+/− 0.88 SD the first day (D00), decreasing along the cycle until it reaches a percentage of 1.24%+/− 0.01 SD D16. Other phyla present in smaller percentages, such as *Patescibacteria, Verrucomicrobiota, Chloroflexi* and *Planctomycetota*, increased during the days. One candidate phylum (WPS-2 = *Eremiobacterota*) and one phylum without identification was found.

At family level differences between days can be observed (Fig. 1B). Day D00 shows singularities comparing to the other three days. Most abundant family at day D00 was *Bacteroidaceae*, with a 6.70%+/− 1.03 SD. This family drastically decreased throughout the crop cycle until D16 with a 0.13% +/− 0.11 SD. Similarly, family *Lachnospiraceae* almost disappeared at the end of the cycle. Family *Xanthomonadaceae* was present in all samples with a similar percentage (4–6%). However, the presence of some families increased during days, such as *Sphingomonadaceae* (from 1.36% +/− 0.47 SD to 7.99% +/− 0.70 SD), *Chitinophagaceae* (from 1.45% +/− 0.34 SD to 5.03% +/− 0.14 SD), *Devosiaceae* (from 0.94% +/− 0.07 SD to 2.86% +/− 0.74 SD) or *Spirosomaceae* (0.20% +/− 0.06 SD to 2.25% +/− 0.20 SD).

Alpha diversity results showed that there were not significant differences among days according to Faith’s phylogenetic diversity (*p* = 0.059) and Pielou’s species evenness (*p* = 0.053). Bacterial community richness and evenness showed significant differences by Shannon’s index (*p* = 0.027). However, results showed higher diversity with-in samples at day 4 (D04) and lower at day zero (D00). Beta diversity results showed significant differences by Bray Curtis: abundance without phylogeny (*p* = 0.001) and unweighted UniFrac distance: presence and absence of OTUs (Operational Transcriptomic Unit) with phylogeny (*p* = 0.001).

An ANCOM test was also carried out, to compare the composition of the microbiome within bacterial populations. Results identified 19 different abundances profiles across samples by days at genus level. *Aquicella, Halomonas, Solitalea, Lacihabitans, Caulobacter* and *Anaerosporobacter* had the highest W value (Supplementary material Table S1).

**Fig. 2:**
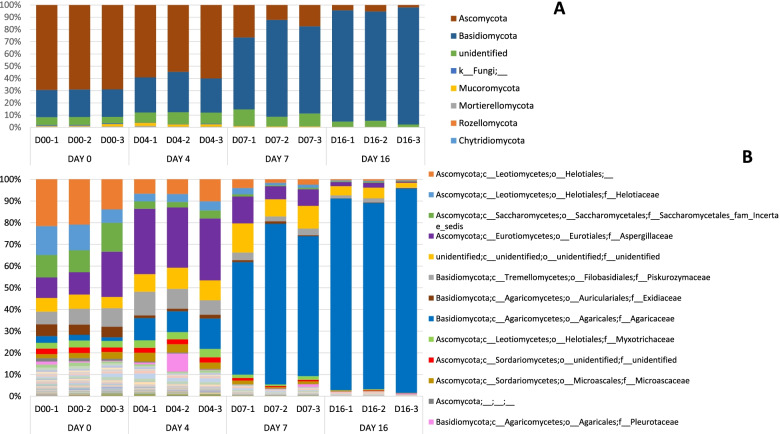
Barplot with the Fungal ITS2 metataxonomical classification. Legend: Fungal ITS2 metataxonomical classification by days at Phylum level (**A**) and at Family level (**B**)

The most frequently identified fungal phyla are *Ascomycota* and *Basidiomycota*. Noteworthy, significant number of sequences were not classified in any phylum (Fig. 2A). At day zero (D00) the most abundant phylum was *Ascomycota* (69.13%+/− 0.27 SD). This trend changes along the cultivation cycle with phylum *Ascomycota* decreasing D04 (57.90%+/− 2.87 SD), D07 (18.68%+/− 7.22 SD) until D16 with 3.86%+/− 1.7 SD. Phylum *Basidiomycota* increases from D00 (22.38%+/− 0.10 SD) until D16 (91.95% +/− 3.29 SD), becoming the dominant phylum from D07. This trend can be also observed at Family level (Fig. 2B), the *Agaricaceae* increasing from D00 (2.44%+/− 0.73 SD) to the D16 (89.43%+/− 4.33 SD).

At family level the change in the populations of the different fungi families has been clearly observed. Family *Agaricaceae* displaced the other families along the crop cycle while *A. bisporus* mycelium is growing in the casing material. In the first samples, *Helotiaceae* family was present in an important percentage (18.74%+/− 4.29 SD) that almost disappears at D16 (0.54%+/− 0.13 SD), as well as other families such as *Piskurozumaceae, Exidiaceae* or *Myxotrichaceae*. On the other hand, the presence of the family *Aspergillaceae* increased from D00 (13.54%+/− 6.32 SD) to the D04 (28.84%+/− 1.23 SD) and then decreased by day D07 and D16 (1.58%+/− 0.94 SD).

Alpha diversity results showed significant differences among days using Faith’s diversity (*p* = 0.024), Pielou’s evenness (*p* = 0.023) and Shannon’s index (*p* = 0.024). Beta diversity results showed also significant differences by Bray Curtis: abundance without phylogeny (*p* = 0.001) and unweighted UniFrac distance (*p* = 0.001).

ANCOM test results identified different abundance across samples by days at genus level in genus *Agaricus* (Supplementary material Table S2).

#### Analysis data-set TREATMENTS D07

**Fig. 3:**
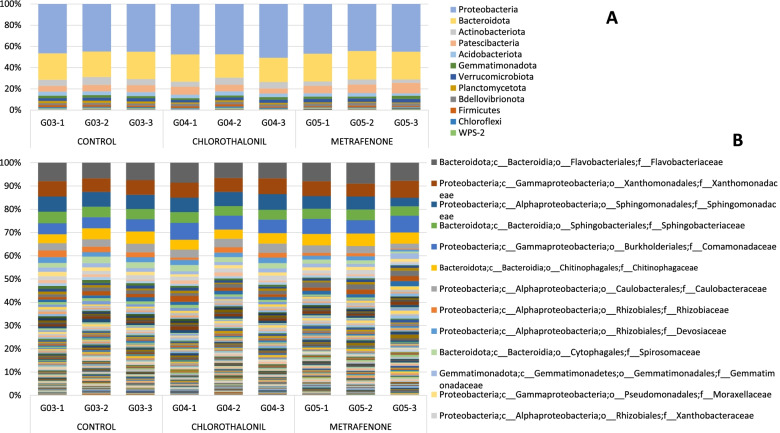
Barplot with the Bacterial 16S metataxonomical classification. Legend: Bacterial 16S metataxonomical classification by treatments at Day 7 at Phylum level (**A**) and at Family level (**B**)

Two different fungicide treatments were applied in the trial on day D04: chlorothalonil and metrafenone. Casing samples taken from a control without any treatment were also analysed. The most representative bacterial phyla were *Proteobacteria* and *Bacteroidota* for all samples in the data-set TREATMENTS D07 (Fig. 3A). The highest *Proteobacteria* abundance corresponds to chlorothalonil treatment with 48.51%+/− 1.84 SD. Three candidate phyla were found, WPS-2, WS2 and FCPU426. Most abundant families are common in the treatments and the control (Fig. 3B), such as *Flavobacteriaceae* (8–9%), *Xanthomonadaceae* (6.7–6.9%) and *Sphingomonadaceae* (6.8–5.3%). Family *Chitinophagaceae* showed higher abundance in metrafenone treatment (5.4% +/−.036 SD) and lower in chlorothalonil treatment (4.51% +/− 0.32 SD). Other families such as *Rhizobiaceae* had a higher abundance in the control (2.64% +/− 0.43 SD) than with the treatments (chlorothalonil 1.90 +/− 0.70 SD and Metrafenone 1.02% +/− 0.54 SD). Family *Rhodanobacteraceae* presence is 1% higher with Metrafenone treatment 2.42% +/− 0.13 SD.

Alpha diversity results showed that there were not significant differences between all treatments using Faith’s diversity (*p* = 0.956), Pielou’s evenness (*p* = 0.875) and Shannon’s index (*p* = 0.956). Beta diversity results showed no significant differences by Bray Curtis: abundance without phylogeny (*p* = 0.149) and unweighted UniFrac distance (*p* = 0.237).

ANCOM test results did not identify different abundances across samples by treatments.

**Fig. 4:**
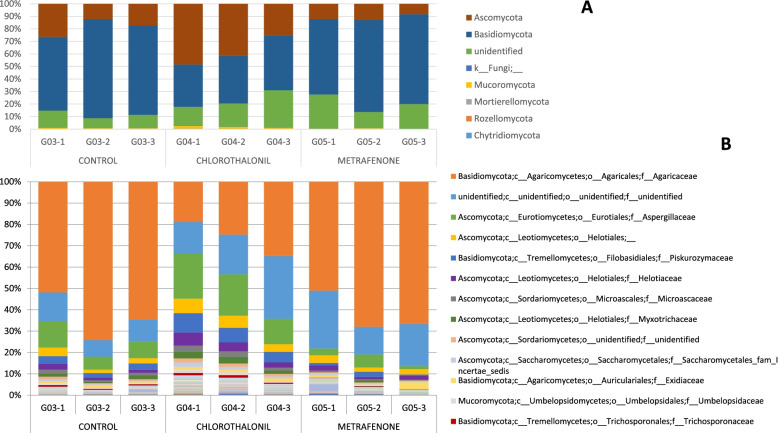
Barplot with the Fungal ITS2 metataxonomical classification. Legend: Fungal ITS2 metataxonomical classification by treatments at Day 7 at Phylum level (**A**) and at Family level (**B**)

For the fungal phylum level (Fig. 4A) in the control without treatment, *Basidiomycota* was the most abundant phylum with 69.71%+/− 10.22 SD. For chlorothalonil treatment both phyla were in similar proportions: *Basidiomycota* 38.55%+/− 4.95 SD and *Ascomycota* 38.33%+/− 11.88 SD. For this treatment phylum *Mucoromycota* was more abundant than in the other 2 treatments. For metrafenone treatment the most abundant phylum was *Basidiomycota* with 68.43%+/− 7.20 SD.

At family level there were around 10–20% of sequences unidentified (Fig. 4B). Family *Agaricaceae* in the control and with metrafenone treatment had a high presence (63.48%+/− 11.15 SD and 61.88%+/− 9.41 SD) while in the chlorothalonil treatment the percentage of this family was just 26.09%+/− 8.04 SD. The other two families identified with an important presence were *Aspergillaceae* and *Piskurozymaceae*.

Alpha diversity results showed non-significant differences between all treatments using Faith’s diversity (*p* = 0.066), Pielou’s evenness (*p* = 0.060) and Shannon’s index (*p* = 0.060). Beta diversity results showed significant differences by Bray Curtis: abundance without phylogeny (*p* = 0.013) and unweighted UniFrac distance (*p* = 0.005).

ANCOM test results do not identify different abundance across samples by treatment at genus level.

#### Analysis data-set TREATMENTS D16

**Fig. 5:**
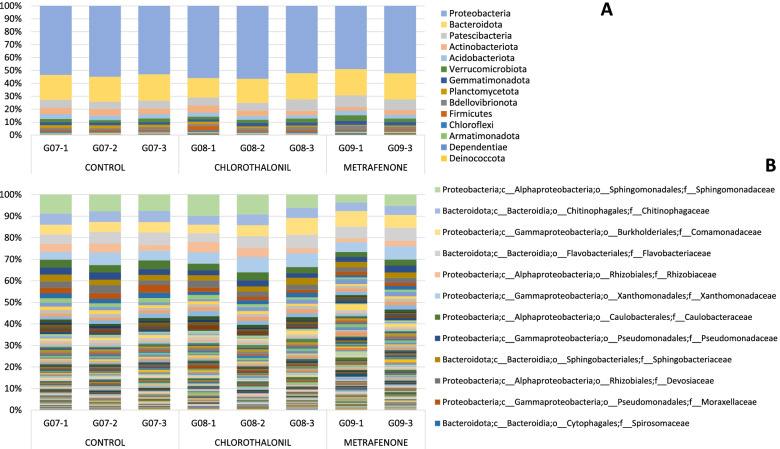
Barplot with the Bacterial 16S metataxonomical classification. Legend: Bacterial 16S metataxonomical classification by treatments at Day 16 at Phylum level (**A**) and at Family level (**B**)

Similar phylum abundance was found for the data-set TREAMENTS D16 (Fig. 5A). *Proteobacteria* phylum also showed the highest presence in all samples, representing more than 50% of the bacteria identified. Likewise, for this data-set, *Proteobacteria* was more abundant in chlorothalonil treatment with 54.78%+/− 2.34 SD. At family level (Fig. 5B) the most common families in all samples were *Sphingomonadaceae* (8.69%+/− 0.84 SD at control, 9.03%+/− 2.26 SD with chlorothalonil and 4.54%+/− 1.29 SD with metrafenone treatment), *Chitinophagaceae, Comamonadaceae, Flavobacteriaceae* and *Rhizobiaceae*.

Alpha diversity showed non- significant differences between all treatments using Faith’s diversity (*p* = 0.235), Pielou’s evenness (*p* = 0.367) and Shannon’s index (*p* = 0.986). Beta diversity results showed significant differences by Bray Curtis: abundance without phylogeny (*p* = 0.019) and unweighted UniFrac distance (*p* = 0.011).

ANCOM test results identified different abundance across samples by treatments at genus level. There were 5 genera identified: *“uncultured*”, *Pirellula, Vicingus, 37–13* and *Sandaracinus* (Supplementary material Table S3).

**Fig. 6:**
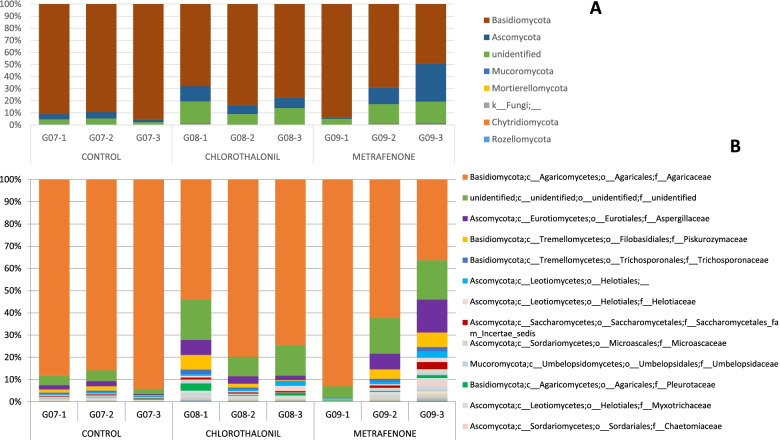
Barplot with the Fungal ITS2 metataxonomical classification. Legend: Fungal ITS2 metataxonomical classification by treatments at Day 16 at Phylum level (**A**) and at Family level (**B**)

In this data-set the fungal phylum *Basidiomycota* was the most abundant in all treatments (Fig. 6A). However, differences in the abundance could be observed. In the case of metrafenone treatment, *Basidiomycota* represented 70.72%+/− 22.34 SD and *Ascomycota* at 15.35%+/− 15.29 SD. These standard deviations were higher than in the other treatments. For chlorothalonil treatment, *Basidiomycota* phylum had a presence of 76.29%+/− 7.91 SD and for the control, *Basidiomycota* was the most abundant with 91.95%+/− 3.29 SD.

At family level *Agaricaceae* was the dominant family (Fig. 6B). However, its presence was lower in the treatments (chlorothalonil 69.37%+/− 13.65SD and metrafenone 63.28%+/− 28.38 SD) comparing to the control (89.43%+/− 4.33 SD). In this data-set there were also a high number of unidentified sequences. Similarly to the data-set TREATMENT D07, other families with an important presence were *Aspergillaceae* and *Piskurozymaceae*.

Alpha diversity results showed no-significant differences between all treatments using Faith’s diversity (*p* = 0.670), Pielou’s evenness (*p* = 0.193) and Shannon’s index (*p* = 0.288). Beta diversity results showed no-significant differences by Bray Curtis: abundance without phylogeny (*p* = 0.324) and unweighted UniFrac distance (*p* = 0.641).

ANCOM test does not identify differences in abundance across samples by treatment at genus level.

## Discussion

The goal of our research is to understand the changes in the microbial dynamics that occur during mycelium development and mushroom (*A. bisporus*) establishment in the casing soil, and the influence of two fungicide treatment applications on the microbial population changes of this active ecosystem.

The fungicide treatments used in this study were chlorothalonil and metrafenone. Chlorothalonil is a wide spectrum fungicide, which belongs to the chloronitrile substance group. It has been used in mushroom industry mainly against *Mycogone perniciosa* and *Lecanicillium fungicola*. Nevertheless, it has been reported to be effective against *Cladobotryum mycophilum* [11, 12] and *Trichoderma* spp. [13], although the selectivity of this active substance is reduced [12]. However, chlorothalonil resistance in *Lecanilicium fungicola* has been reported [14]. The use of chorothalonil is no longer allowed in Europe, but it is in North America, together with thiabendazole and thiophanate-methyl [15]. In Europe just metrafenone and prochloraz are approved for application in white button mushroom production [16]. Metrafenone belongs to the substance group of benzophenone and is used primarily in cereal production. The mode of action of metrafenone interferes with hyphal morphogenesis [17], and in mushroom industry has been used against *Cladobotryum mycophilum* [12]*.*

The structure of the populations of both bacteria and fungi, at the beginning of the crop cycle (day zero = D00), fluctuate with respect to the following days. As reported in our previous project [18] data analysed confirm that the casing soil in contact with the compost colonized by *A. bisporus*, undergoes changes in the dynamics of its population structures. In agreement with other research on *A. bisporus,* we observed that the predominant bacterial phylum identified in all the casing soil samples was *Proteobacteria*, followed by *Bacteroidota* [19, 20]. *Proteobacteria* population increased along the days as seen in other studies [21], and *Firmicutes* phylum decreased throughout the cycle. Other phyla present in smaller percentages, such as *Patescibacteria, Verrucomicrobiota, Chloroflexi* and *Planctomycetota*, increased during the days. Most abundant family at day D00 was *Bacteroidaceae*, which decreased deeply during the cycle, like family *Lachnospiraceae* that almost disappeared at the end of the cycle. However, the presence of some families increased during days, such as *Sphingomonadaceae, Chitinophagaceae*, *Devosiaceae* or *Spirosomaceae*. Results identified different abundances at genus level across samples by days in 19 genera. The genera with highest W value were *Aquicella, Halomonas, Solitalea, Lacihabitans, Caulobacter* and *Anaerosporobacter*. These genera were not present at day D00 and they started to grow to significant populations during the incubation. Between day D07 and D16 a change in family’s abundance was observed. *Flavocteriaceae* and *Xanthomonadaceae* had a higher presence at day D07 while *Sphingomonadaceae* and *Chitinophagaceae* at day D16. At genus level, *Flavobacterium* was the most abundant in all samples and *Pseudomonas* increase along days, as has been already published in our previous research [18]. Five genera with different abundance have been identified between treatments at D16: *“uncultured”, Pirellula, Vicingus, 37–13* (unclassified *Bacteroidetes*) and *Sandaracinus*. Genus *Pirellula* seems to be dominant during pinning [22] and during the first flush in compost samples [23]. These results also confirm the chlorothalonil effect on soil bacterial [24], on several groups such as *Flavobacterium (Vicingus)* and *δ-proteobacteria (Sandaracinus)*. The most abundant bacterial species identified (Supplementary material Fig. 9) at day D00 was *Bacteroides graminisolvens*, a xylanolytic anaerobe bacterial strain [25] that could be related with peat formation in natural peatland since it takes place in waterlogged soils with little or no access to oxygen. This genus almost disappears along the incubation days.

At day D00 the most abundant fungal phylum was *Ascomycota,* but throughout the cultivation cycle, the phylum *Basidiomycota* became dominant as described previoustly [26, 27]. As aforementioned, *A. bisporus* replaced the other species and colonized the casing soil material. For this reason, from day D16 it becomes very difficult to determine the presence or changes in the populations of other fungi, since reads other than *Agaricus/Basidiomycota* are residual. Families *Helotiaceae*, *Piskurozymaceae, Exidiaceae* or *Myxotrichaceae* were present in an important percentage at D00 and almost disappeared at D16. Other studies found abundance of genus *Agaricus*, *Apiotrichum, Meliniomyces, Mycothermus, Candida* and *Pseudeurotium* [28]. At species level (Supplementary material Fig. 12) we found the presence of *Agaricus*, *Apiotrichum, Meliniomyces* and *Candida,* among others, but not *Meliniomyces* or *Mycothermus*. On the other hand, *Aspergillaceae* family presence increased from day D00 to day D04 and then it decreased at days D07 and day D16. At day D07 for the control without treatment, *Basidiomycota* was the most abundant phylum while for chlorothalonil treatment both *Basidiomycota* and *Ascomycota* showed similar abundance. In chlorothalonil treatment, phylum *Mucoromycota* was more abundant than in control and metrafenone. In metrafenone treatment, the most abundant phylum was *Basidiomycota*. Family *Agaricaceae* in control and with Metrafenone treatment had a high presence while in chlorothalonil treatment the presence of this family is lower, something that could be associated to the reduced selectivity of this active substance which provokes a delay in the colonisation of the casing by the host [12]. The other two families identified with an important presence were *Aspergillaceae* and *Piskurozymaceae*. At D07 we observed an effect of chlorothalonil treatment on the development of *A. bisporus*, being lower its presence in the chlorothalonil treated samples than in the others (Supplementary material Fig. 13). The toxic effect of chlorothalonil to mushrooms when incorporated into the casing has been reported since 1978 and is confirmed by our results [12, 22, 24]. Beta diversity results corroborated fungicide treatments effect on the bacterial and fungi populations. At D16 phylum *Basidiomycota* was the most abundant in all treatments. Metrafenone treatment had higher standard deviations than other treatments. At family level *Agaricaceae* was the dominant family, with a remarkable higher presence in the control. Reads associated to the presence of fungal diseases were detected in the samples as reported in previous works [18, 26]. *Lecanicillium* genus was detected in samples at D00 and D04, but also in one sample at D07, treated with chlorothalonil. A small number of sequences belonging to *Mycogone perniciosa* were found just in one sample at D00. The presence of mycoparasites in raw materials (D00) can be associated to a contamination prior to the application in crop. This is very relevant since an early infection can provoke important damages due to heavy disease symptoms.

For the metataxonomy analysis with fungal ITS2, many sequences were assigned to “unidentified” taxon. These lack of results was also found in other similar studies [22, 29] although they were focused on the compost dynamics and not the casing soil changes. In the casing layer unidentified OTUs were also detected in our previous study, mostly in the raw material [26]. Unfortunately, through metataxonomic analysis is not always possible to reach species level, only some sequences of bacteria and fungi have been assigned to the level of species. There is still much work to do in taxonomic identification to build better and more complete databases.

## Conclusions

The aim of the study was to understand the changes experienced by different populations of fungi and bacteria in the casing soil during button mushroom production in order to improve peat microbial knowledge and find more sustainable alternatives to peat-based casing. This study shows that the use of QIIME2 is valid for NGS sequence analysis in this type of samples. Noteworthy, available resources in databases do not allow metataxonomic studies to reach the species level in most cases, just the genus level which is a limitation for the depth of the analysis. Significant differences have been found between the populations of both fungi and bacteria between the days of mycelium incubation, being more pronounced between the casing day (D00) and day (D04). Differences have also been found between the fungicide treatments applied. Understanding the effect of chemical treatments on the microbiome equilibrium in the casing can help to design more specific and safe fungicides to cope with mushroom pathogens. More detailed studies are also required to explore the relationship between microbial activity and diversity in casing soil and its implications in the mycelium and primordia development. Finally, a more thorough understanding of the pathogens control would have the potential to increase the quality and quantity of mushrooms produced.

## Methods

### Casing sampling

Cultivation room was filled with phase III compost from a commercial mushroom compost yard (Germinados de Lodosa, L.S., Lodosa, Spain). The *A. bisporus* strain used was Sylvan A15. The casing soil used was 50% black peat and 50% blond peat from Valimex S.L (Andosilla, Spain). Casing pH was adjusted to 8 with limestone addition by the provider. Three biological replicates of the casing samples were destructively taken from the cultivation room along the casing incubation process, from the peat-based casing at four different days (Table 2): casing day (D00), fungicides application day (D04), ruffling day (D07) and at beginning of first flush (D16). A randomized crop design in which fungicide treatments were applied in separated 1m^2^ areas was set up (three replicates per treatment). Chlorothalonil (Banko Champiñón, chlorothalonil 50%, Arysta Lifescience Spain) treatment concentration was 2 ml/m^2^ and metrafenone (Vivando, metrafenone 50%, Basf) treatment application was 0.5 ml/m^2^.

**Table 2:** Samples utilized in the present work

Cod.	Date	Samples
G1	14/01/2019	Casing soil mix at casing stage (50% blond peat, 50% black peat)
G2	18/01/2019	Casing at day 4 at incubation phase^a^
G3	21/01/2019	Casing at day 7 at ruffing (control)
G4	21/01/2019	Casing at day 7 at ruffling (chlorothalonil)
G5	21/01/2019	Casing at day 7 at ruffling (metrafenone)
G7	30/01/2019	Casing at day 16 beginning 1st flush (control)
G8	30/01/2019	Casing at day 16 beginning 1st flush (chlorothalonil)
G9	30/01/2019	Casing at day 16 beginning 1st flush (metrafenone)

^a^Samples at day 4 were taken before fungicide application

### Total DNA extraction

Three biological replicates of the casing samples were studied by extracting genomic DNA (*n* = 3 replicates per sample type). Fresh samples were homogenized in a ceramic mortar with liquid nitrogen. DNA was extracted from up to 0.5 g of casing with NucleoSpin® Soil kit (MACHEREY-NAGEL). DNA quantity and quality were checked using 2 μl of the purified template in a Qubit 2.0 Fluorometer and the Qubit dsDNA BR Assay kit (Thermo Fisher Scientific, MA, USA) and finally it was visualized on a 1.5% agarose gel stained with Midori-Green Advance staining (Nippon Genetics, Tokyo, Japan).

### PCR amplification

Library preparation for 16S rRNA and ITS2 gene amplicon sequencing was performed separately following the Illumina (San Diego, CA, USA) recommendations with some modifications. A 2-step amplification procedure was used [30] using paired end universal bacterial primers [31] for the V3-V4 hypervariable region of the 16S rRNA gene. For the hypervariable region ITS2, the primers ITS3 and ITS4 were used [32]. The primers used contained the Illumina sequencing adapters (overhang nucleotide sequences) added to the gene-specific sequences as described in previous research [26].

### Sequence analyses

Sequencing data were demultiplexed using Illumina bcl2fastq© program. Demultiplexed paired FASTQ sequences were imported in QIIME2 artifact format and analysed with QIIME2 v2020.8. Used workflow is described in detail in the following link: https://github.com/Marylou8/Metataxonic-analysis-using-Qiime2-workflow. Quality control was carried out using the DADA2 pipeline [33] incorporated into QIIME2 [34, 35]. The DADA2 program filtered out PhiX reads, removed chimeric sequences and assigned reads into Amplicon Sequence Variants (ASVs). Taxonomic annotation for bacteria was obtained using SILVA v138 database [36]. Taxonomic annotation for fungi was obtained using UNITE v8.2 2020 database [37]. Chloroplast and mitochondrial contaminants were detected and filtered using the QIIME2 “taxa filter-table” and “taxa filter-seqs” commands.

To filter out low-abundance features, we follow the approach of Morton (https://forum.qiime2.org/t/ancom-giving-strange-w-values/1002/11) where those features which do not sum at least 10 sequences among all samples, as well as those that only appear in one sample were filter out (command “feature-table filter-features”). Differential abundance analysis was analysed with ANCOM test [38]. The data-set will be submitted to National Center for Biotechnology Information (NCBI).

### Statistics

Sequencing statistical analyses were done using QIIME2 v2020.8 [35]. Alpha diversity (within-samples) was analysed using Faith’s phylogenetic diversity: bacterial community richness that incorporates phylogenetic relationships between taxa [39], Pielou’s species evenness: bacterial community evenness [40] and Shannon’s index: bacterial community richness and evenness [41]. To determine significance in alpha diversity, non-parametric Kruskal-Wallis comparisons were performed [42]. Box-and-whisker plots for species richness and evenness were generated using QIIME2. Alpha rarefaction curves were analysed, to assess if sampling depth was enough to observe the full community diversity [43]. Beta diversity was analysed using Bray–Curtis distance (abundance without phylogeny) [44] and unweighted UniFrac distance (presence and absence of OTUs with phylogeny) [45]. Principle Coordinate Analysis (PCoA) plots were generated from Bray-Curtis distances and unweighted UniFrac distance using QIIME2. To generate taxonomy heatmaps R-Studio Version 1.3.1093 was used [46]. Two libraries were necessary to plot the heatmaps from QIIME2 artifacts “tidyverse” [47] and “qiime2R” [48].

## Supplementary Information

**Additional file 1: Table S1.** ANCOM Statistical analysis on the bacteria population with the significant genus data-set DAYS.

**Additional file 2: Table S2.** ANCOM Statistical analysis on the fungi population with the significant genus data-set DAYS.

**Additional file 3: Table S3.** ANCOM Statistical analysis on the bacteria population with the significant genus data-set TREATMENTS-D16.

**Additional file 4: Supplementary Figure 1.** Diversity significant boxplot for 16S analysis by DAY. Right to left: D00, D04, D07 and D16. With Faith Phylodiversity (1A), Pielou’s species evenness (1B) and Shannon’s index (1C). Three-dimensional PCoA visualized using Emperor, built using the Bray Curtis distance matrix (1D) and the unweighted UniFrac distance matrix (1E).

**Additional file 5: Supplementary Figure 2.** Diversity significant boxplot for 16S analysis by TREATMENT. Right to left: Chlorothalonil, Control and Metrafenone. With Faith Phylodiversity (2A), Pielou’s species evenness (2B) and Shannon’s index (2C). Three-dimensional PCoA visualized using Emperor, built using the Bray Curtis distance matrix (2D) and the unweighted UniFrac distance matrix (2E).

**Additional file 6: Supplementary Figure 3.** Diversity significant boxplot for 16S analysis by TREATMENT. Right to left: Chlorothalonil, Control and Metrafenone. With Faith Phylodiversity (3A), Pielou’s species evenness (3B) and Shannon’s index (3C). Three-dimensional PCoA visualized using Emperor, built using the Bray Curtis distance matrix (3D) and the unweighted UniFrac distance matrix (3E).

**Additional file 7: Supplementary Figure 4.** Diversity significant boxplot for ITS2 analysis by DAY. Right to left: D00, D04, D07 and D16. With Faith Phylodiversity (4A), Pielou’s species evenness (4B) and Shannon’s index (4C). Three-dimensional PCoA visualized using Emperor, built using the Bray Curtis distance matrix (4D) and the unweighted UniFrac distance matrix (4E).

**Additional file 8: Supplementary Figure 5.** Diversity significant boxplot for ITS2 analysis by TREATMENT. Right to left: Chlorothalonil, Control and Metrafenone. With Faith Phylodiversity (5A), Pielou’s species evenness (5B) and Shannon’s index (5C). Three-dimensional PCoA visualized using Emperor, built using the Bray Curtis distance matrix (5D) and the unweighted UniFrac distance matrix (5E).

**Additional file 9: Supplementary Figure 6.** Diversity significant boxplot for ITS2 analysis by TREATMENT. Right to left: Chlorothalonil, Control and Metrafenone. With Faith Phylodiversity (6A), Pielou’s species evenness (6B) and Shannon’s index (6C). Three-dimensional PCoA visualized using Emperor, built using the Bray Curtis distance matrix (6D) and the unweighted UniFrac distance matrix (6E).

**Additional file 10: Supplementary Figure 7.** Rarefaction plots of all 23 samples for 16S analysis (sample 26 excluded). 7A: Rarefaction curves (Shannon’s index on Y axis), 7B: Rarefaction curves (Number of observed features on Y axis).

**Additional file 11: Supplementary Figure 8.** Rarefaction plots of all 24 samples for ITS2 analysis. 8A: Rarefaction curves (Shannon’s index on Y axis), 8B: Rarefaction curves (Number of observed features on Y axis).

**Additional file 12: Supplementary Figure 9.** Heatmap with the bacterial taxonomy at Species level of data-set DAYS.

**Additional file 13: Supplementary Figure 10.** Heatmap with the bacterial taxonomy at Species level of data-set TREATMENTS D07.

**Additional file 14: Supplementary Figure 11.** Heatmap with the bacterial taxonomy at Species level of data-set TREATMENTS D16.

**Additional file 15: Supplementary Figure 12.** Heatmap with the fungal taxonomy at Species level of data-set DAYS.

**Additional file 16: Supplementary Figure 13.** Heatmap with the fungal taxonomy at Species level of data-set TREATMENTS D07.

**Additional file 17: Supplementary Figure 14.** Heatmap with the fungal taxonomy at Species level of data-set TREATMENTS D16.

## Data Availability

The datasets analysed during the current study are available in the NCBI SRA repository, with the BioProject accession number PRJNA772891, https://www.ncbi.nlm.nih.gov/sra/PRJNA772891.

## Abbreviations

ASV: Amplicon Sequence Variant
ANCOM: Analysis of Composition of Microbiomes
DADA: Divisive Amplicon Denoising Algorithm
NCBI: National Center for Biotechnology Information
NGS: Next Generation Sequencing
OTU: Operational Transcriptomic Unit
PCR: Polymerase chain reaction
PCoA: Principal Coordinates Analysis
QIIME2: Quantitative Insights Into Microbial Ecology version 2
SD: Standard Deviation
