# Supplementary figures and images for: Influence of Agaricus bisporus establishment and fungicidal treatments on casing soil metataxonomy during mushroom cultivation
Source: BMC Genomics. 2022 Jun 15;23:442. doi: 10.1186/s12864-022-08638-x (PMC9199190; doi:10.1186/s12864-022-08638-x)

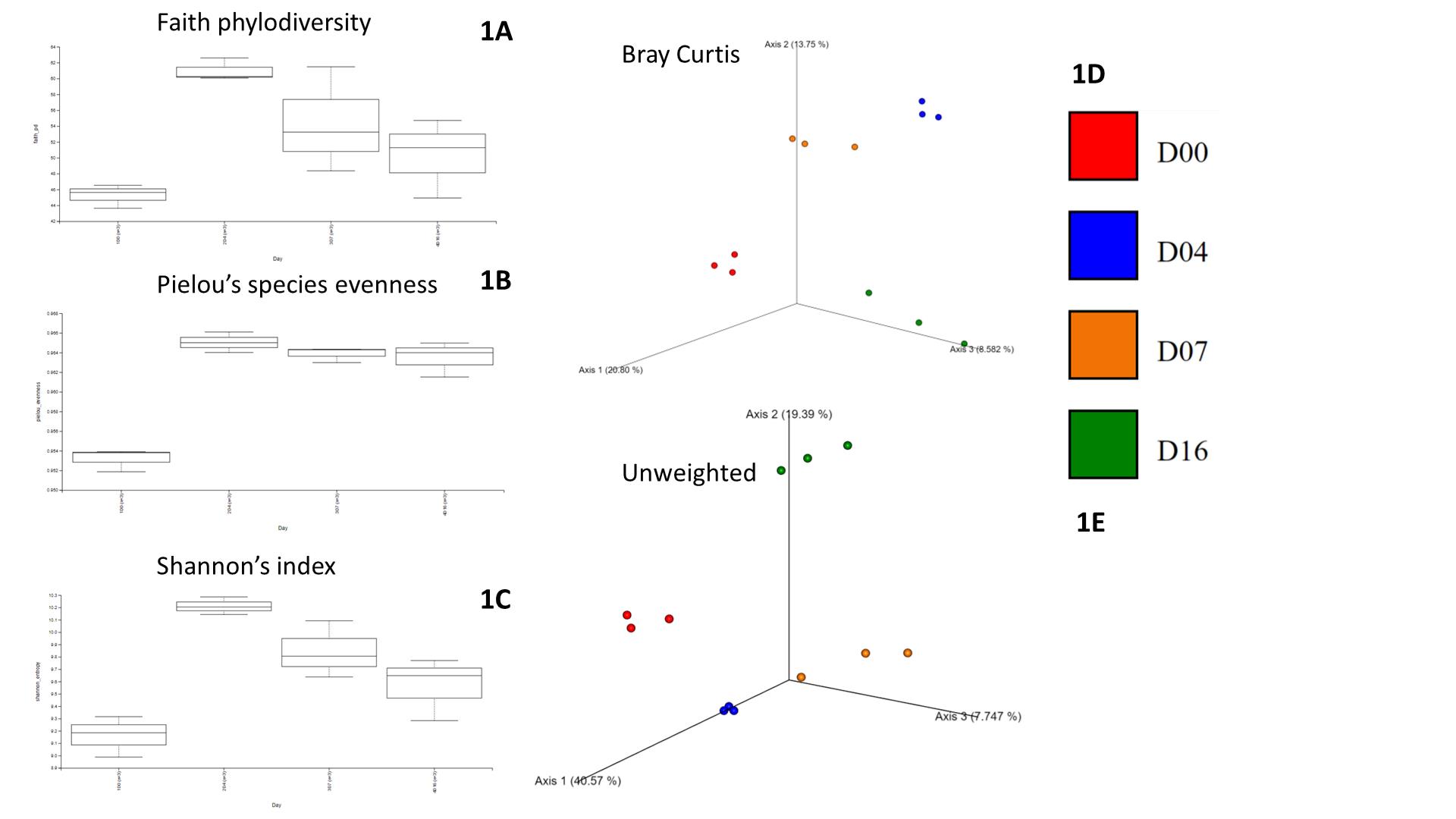

Supplement: Supplementary file 4 — Additional file 4: Supplementary Figure 1. Diversity significant boxplot for 16S analysis by DAY. Right to left: D00, D04, D07 and D16. With Faith Phylodiversity (1A), Pielou’s species evenness (1B) and Shannon’s index (1C). Three-dimensional PCoA visualized using Emperor, built using the Bray Curtis distance matrix (1D) and the unweighted UniFrac distance matrix (1E). [file 12864_2022_8638_MOESM4_ESM.jpg]

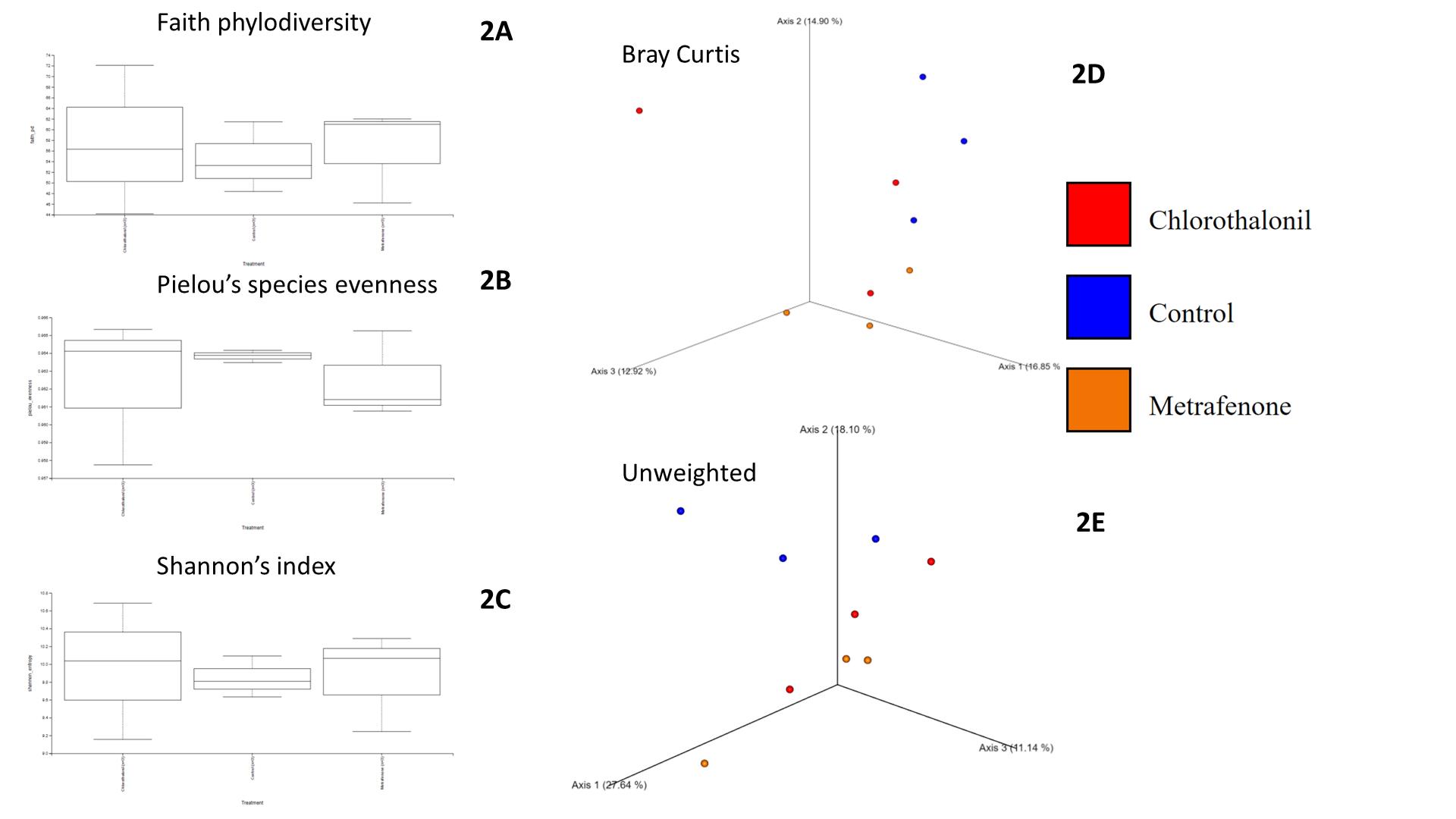

Supplement: Supplementary file 5 — Additional file 5: Supplementary Figure 2. Diversity significant boxplot for 16S analysis by TREATMENT. Right to left: Chlorothalonil, Control and Metrafenone. With Faith Phylodiversity (2A), Pielou’s species evenness (2B) and Shannon’s index (2C). Three-dimensional PCoA visualized using Emperor, built using the Bray Curtis distance matrix (2D) and the unweighted UniFrac distance matrix (2E). [file 12864_2022_8638_MOESM5_ESM.jpg]

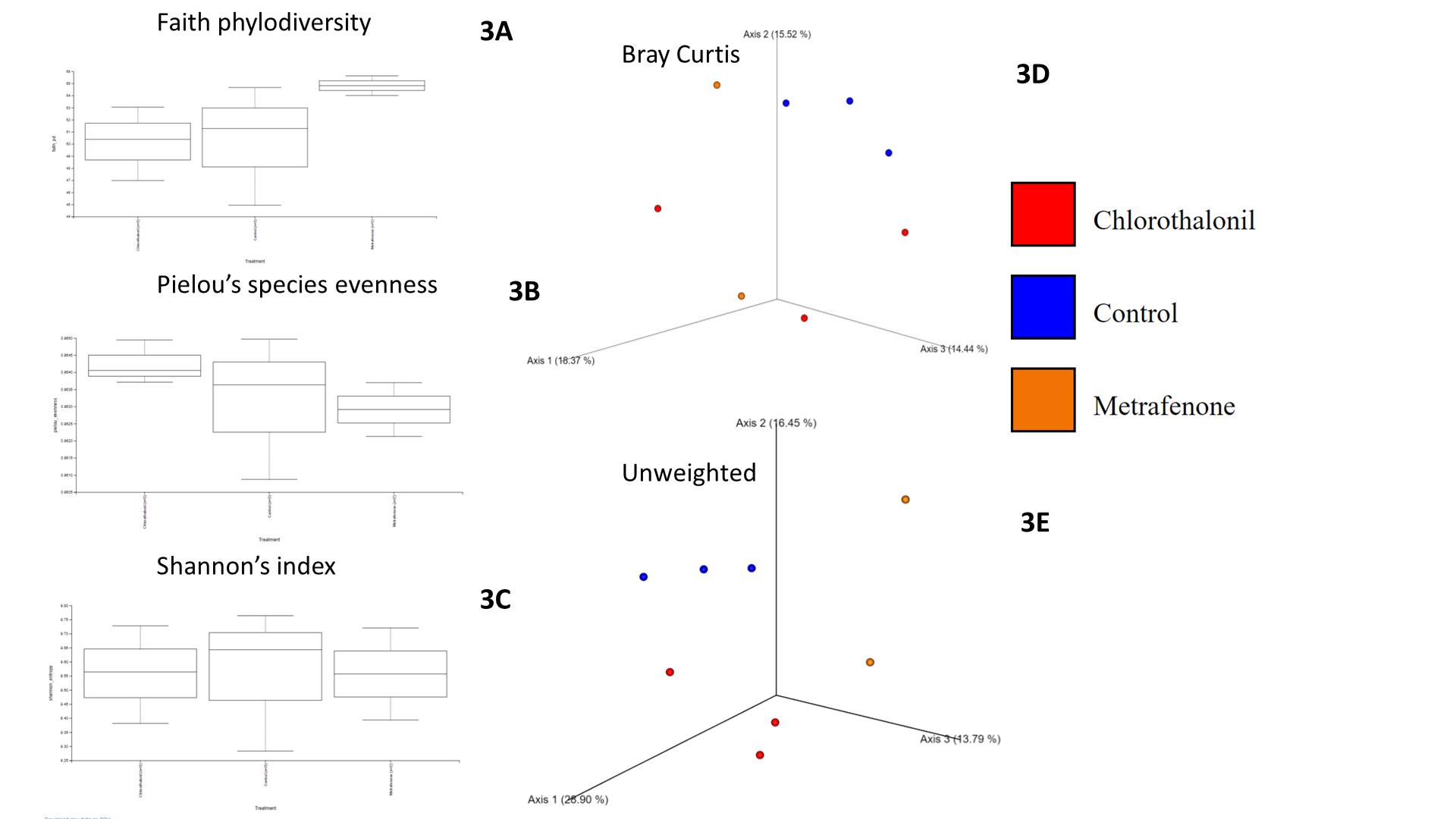

Supplement: Supplementary file 6 — Additional file 6: Supplementary Figure 3. Diversity significant boxplot for 16S analysis by TREATMENT. Right to left: Chlorothalonil, Control and Metrafenone. With Faith Phylodiversity (3A), Pielou’s species evenness (3B) and Shannon’s index (3C). Three-dimensional PCoA visualized using Emperor, built using the Bray Curtis distance matrix (3D) and the unweighted UniFrac distance matrix (3E). [file 12864_2022_8638_MOESM6_ESM.jpg]

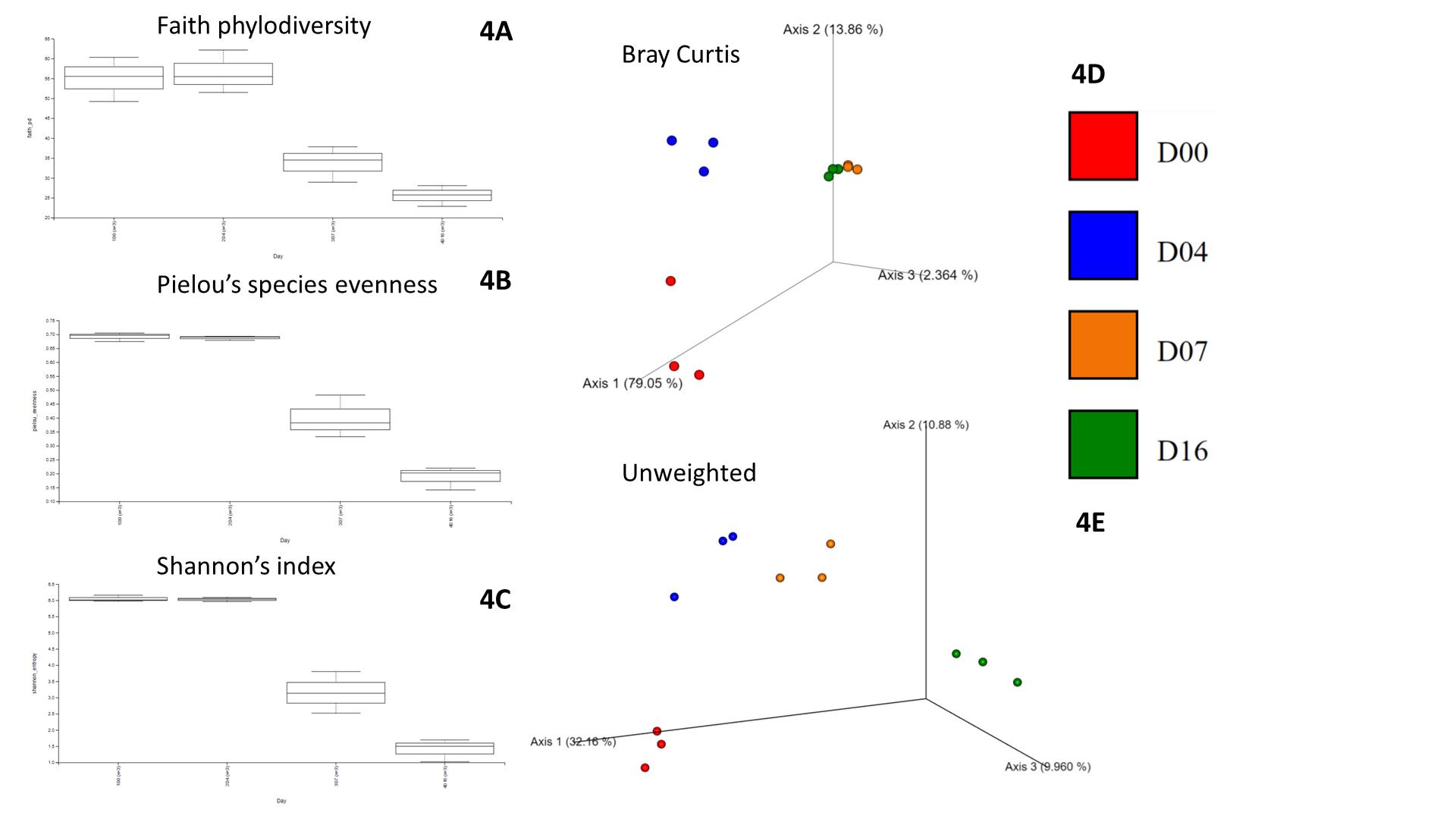

Supplement: Supplementary file 7 — Additional file 7: Supplementary Figure 4. Diversity significant boxplot for ITS2 analysis by DAY. Right to left: D00, D04, D07 and D16. With Faith Phylodiversity (4A), Pielou’s species evenness (4B) and Shannon’s index (4C). Three-dimensional PCoA visualized using Emperor, built using the Bray Curtis distance matrix (4D) and the unweighted UniFrac distance matrix (4E). [file 12864_2022_8638_MOESM7_ESM.jpg]

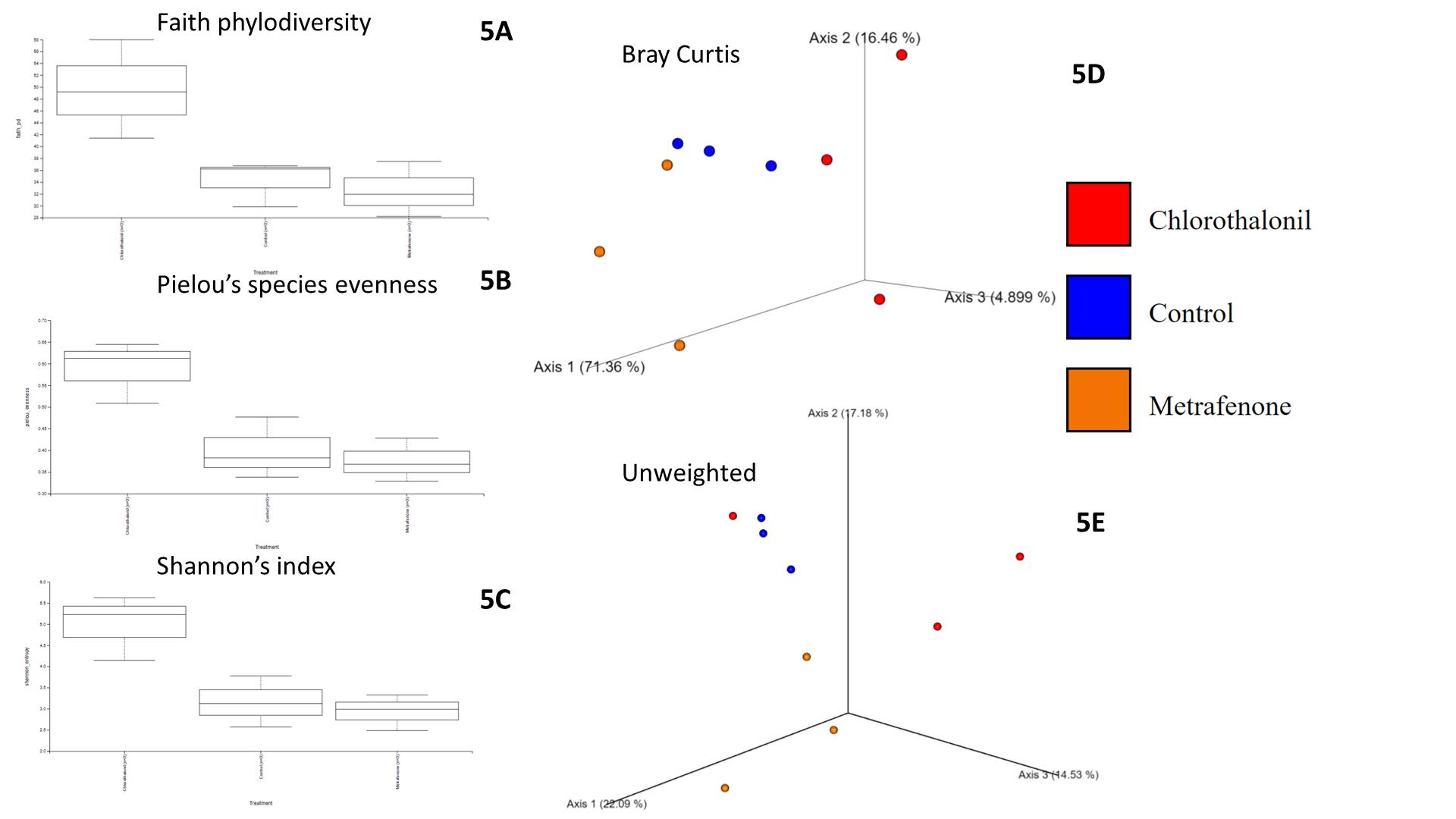

Supplement: Supplementary file 8 — Additional file 8: Supplementary Figure 5. Diversity significant boxplot for ITS2 analysis by TREATMENT. Right to left: Chlorothalonil, Control and Metrafenone. With Faith Phylodiversity (5A), Pielou’s species evenness (5B) and Shannon’s index (5C). Three-dimensional PCoA visualized using Emperor, built using the Bray Curtis distance matrix (5D) and the unweighted UniFrac distance matrix (5E). [file 12864_2022_8638_MOESM8_ESM.jpg]

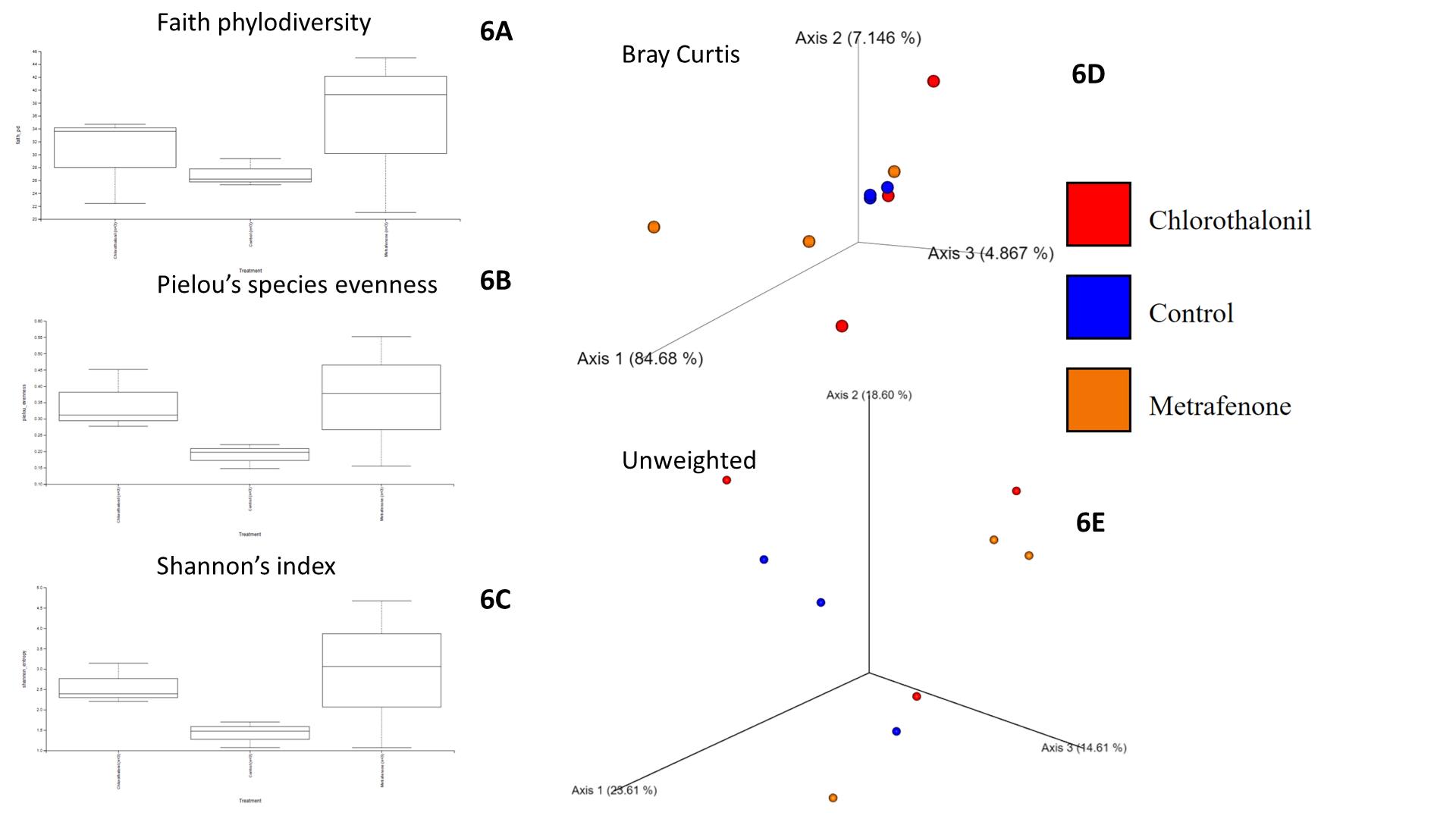

Supplement: Supplementary file 9 — Additional file 9: Supplementary Figure 6. Diversity significant boxplot for ITS2 analysis by TREATMENT. Right to left: Chlorothalonil, Control and Metrafenone. With Faith Phylodiversity (6A), Pielou’s species evenness (6B) and Shannon’s index (6C). Three-dimensional PCoA visualized using Emperor, built using the Bray Curtis distance matrix (6D) and the unweighted UniFrac distance matrix (6E). [file 12864_2022_8638_MOESM9_ESM.jpg]

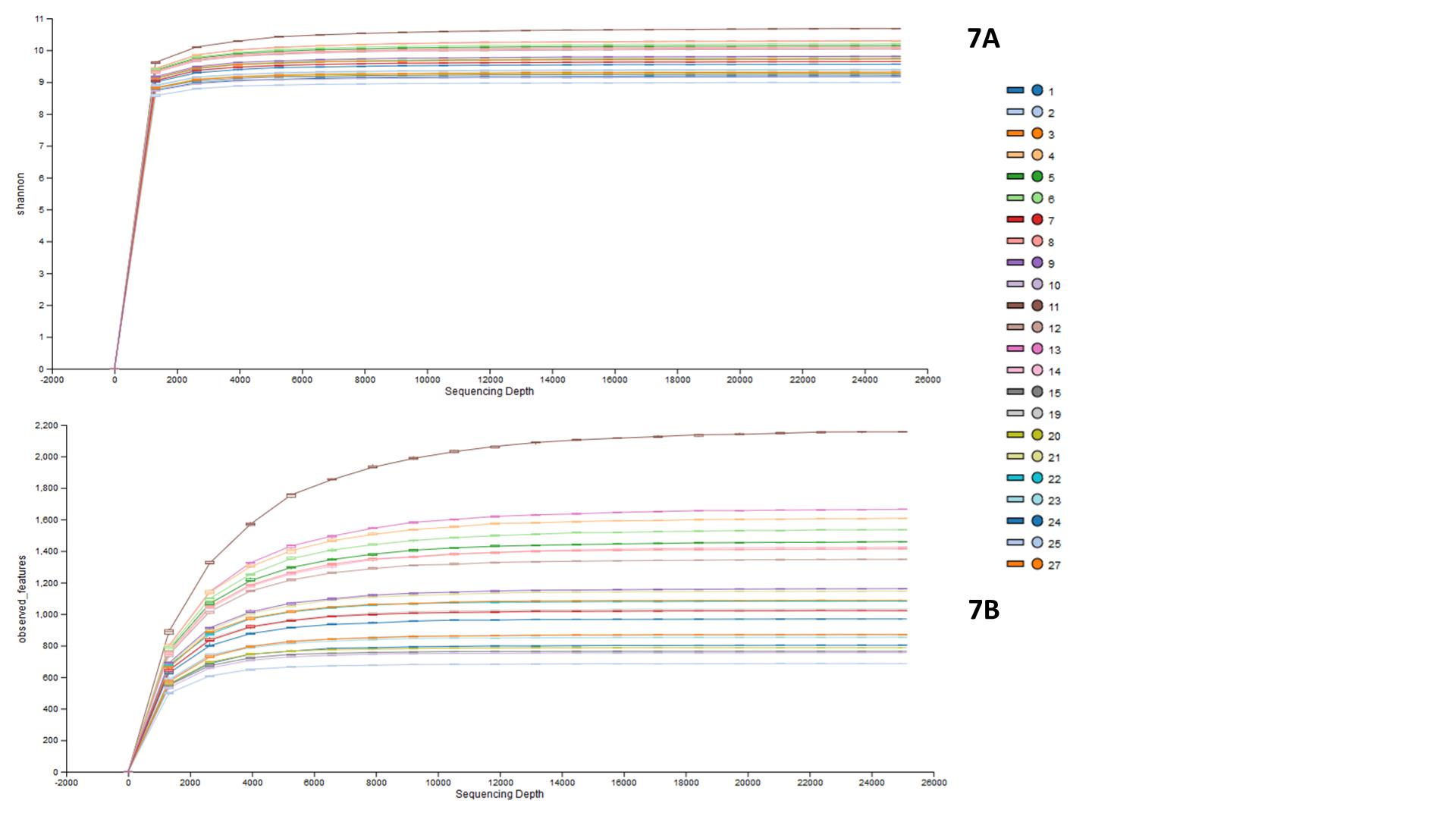

Supplement: Supplementary file 10 — Additional file 10: Supplementary Figure 7. Rarefaction plots of all 23 samples for 16S analysis (sample 26 excluded). 7A: Rarefaction curves (Shannon’s index on Y axis), 7B: Rarefaction curves (Number of observed features on Y axis). [file 12864_2022_8638_MOESM10_ESM.jpg]

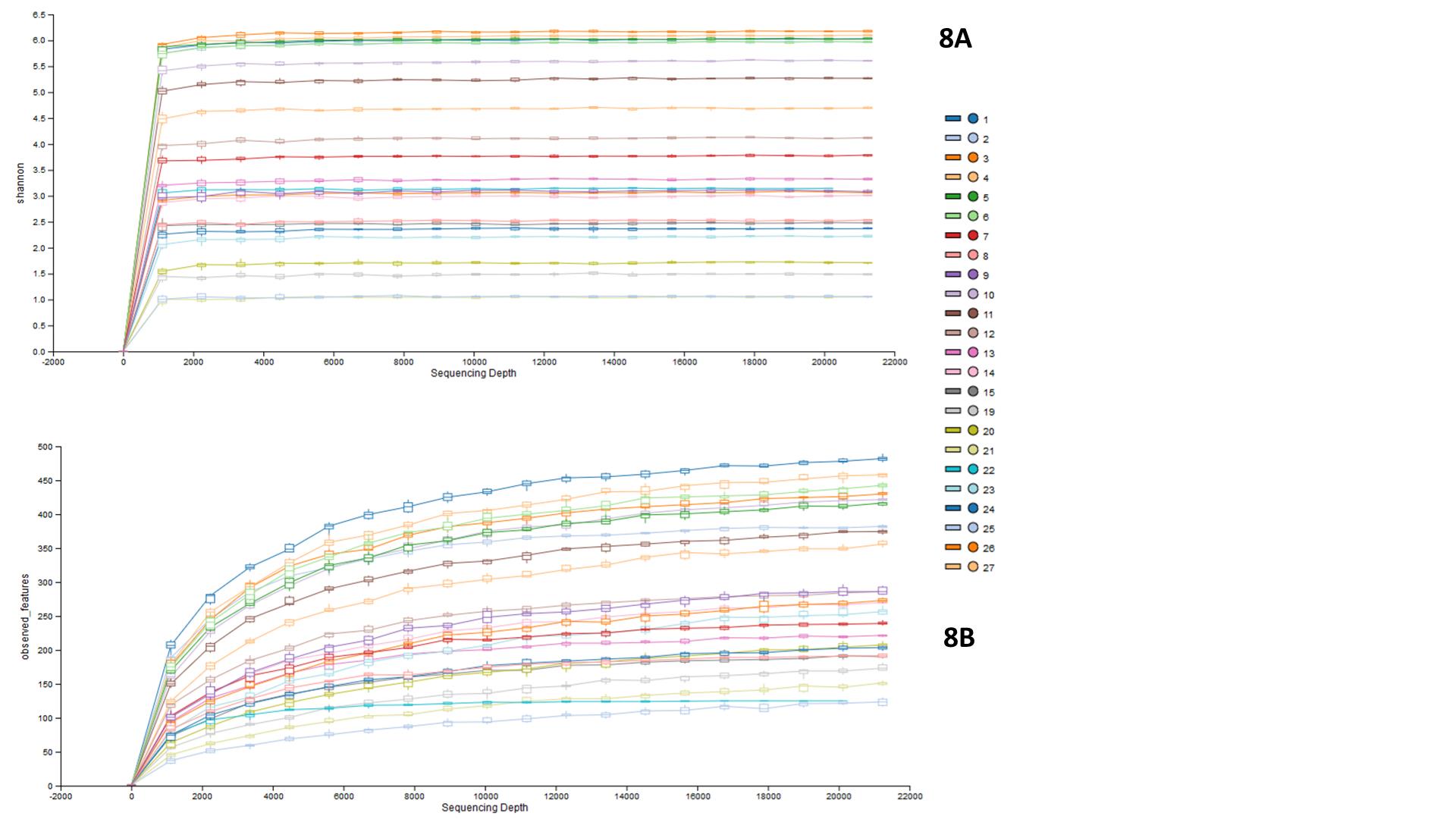

Supplement: Supplementary file 11 — Additional file 11: Supplementary Figure 8. Rarefaction plots of all 24 samples for ITS2 analysis. 8A: Rarefaction curves (Shannon’s index on Y axis), 8B: Rarefaction curves (Number of observed features on Y axis). [file 12864_2022_8638_MOESM11_ESM.jpg]

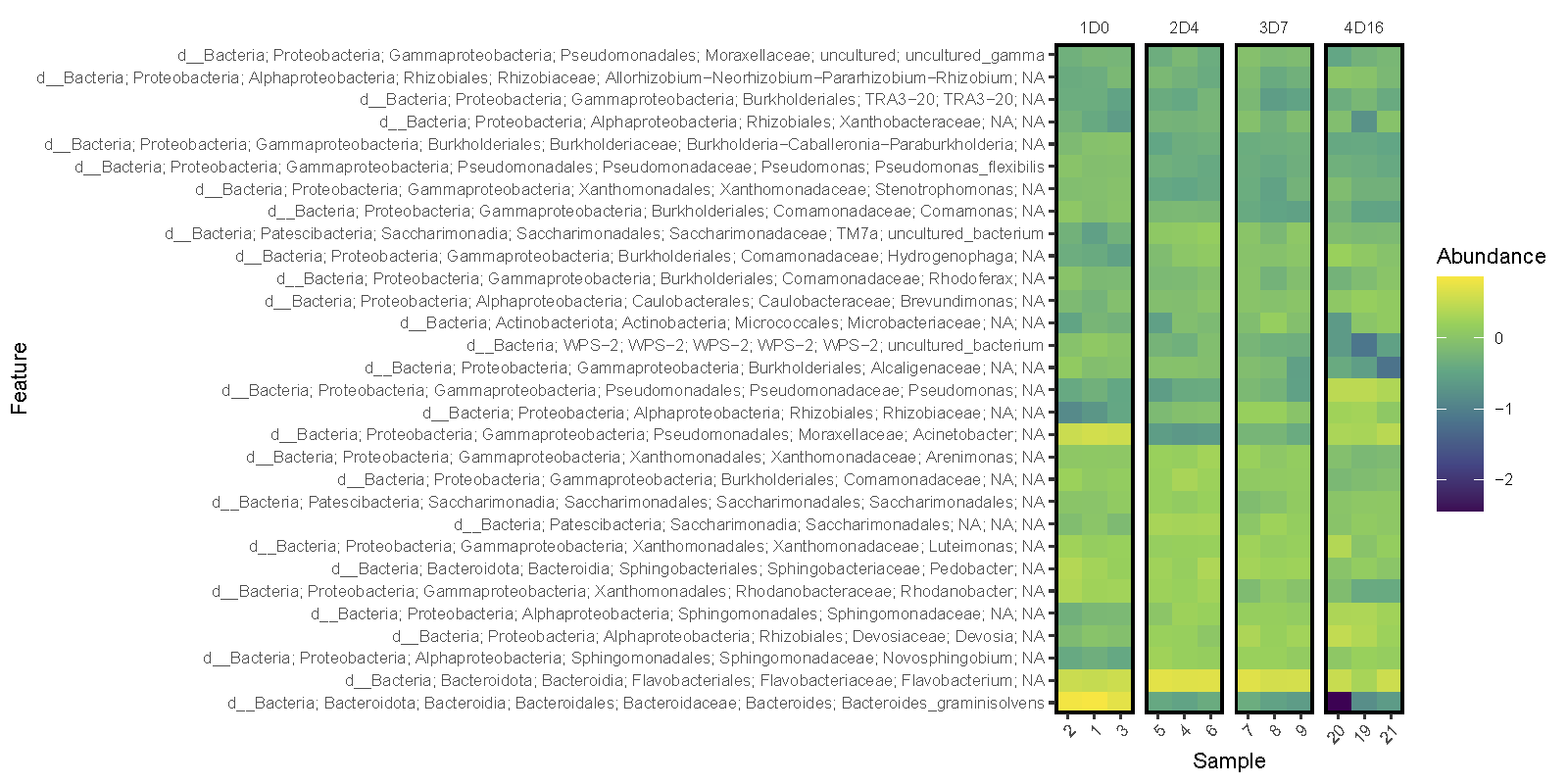

Supplement: Supplementary file 12 — Additional file 12: Supplementary Figure 9. Heatmap with the bacterial taxonomy at Species level of data-set DAYS. [file 12864_2022_8638_MOESM12_ESM.tiff]

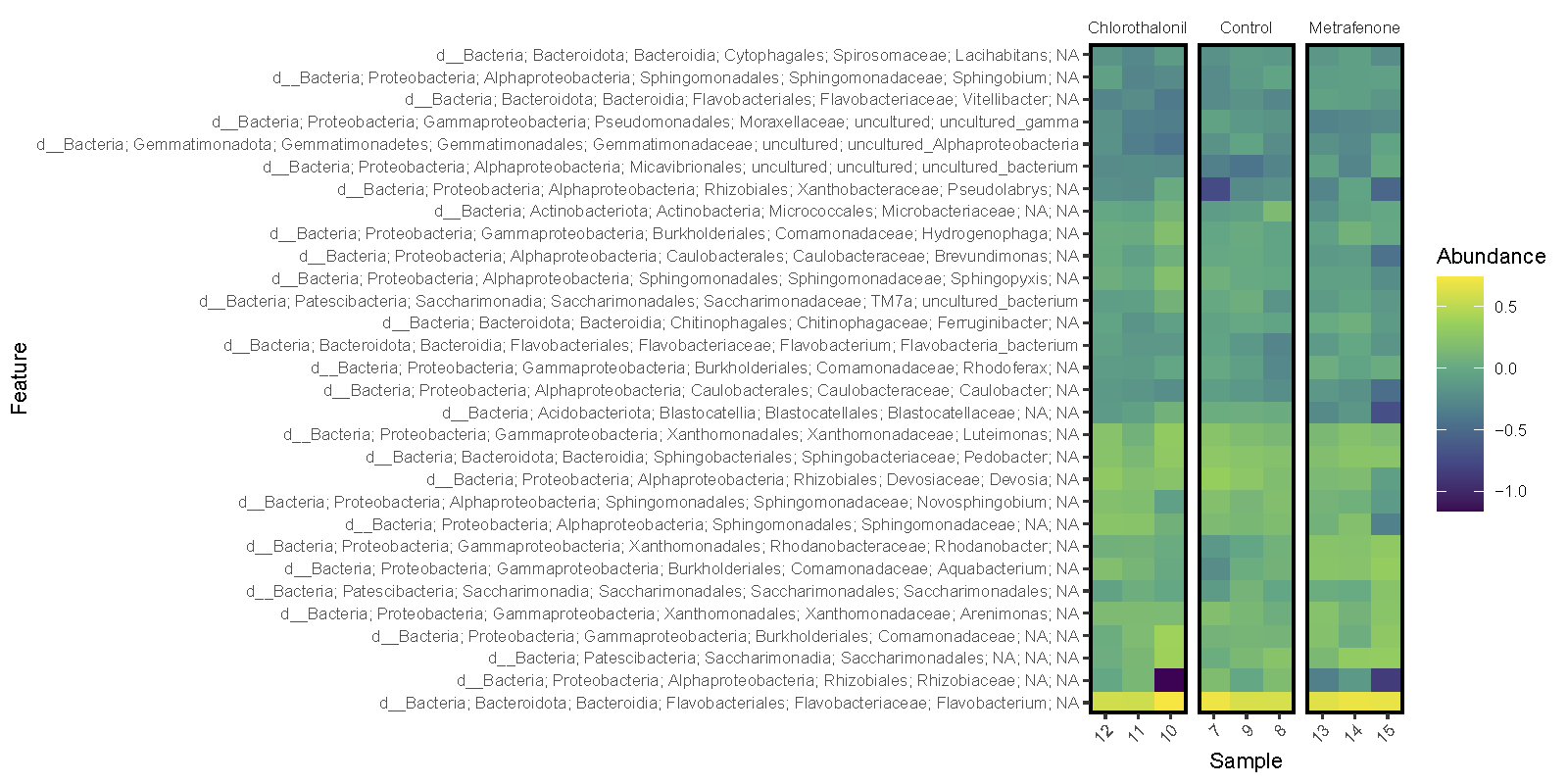

Supplement: Supplementary file 13 — Additional file 13: Supplementary Figure 10. Heatmap with the bacterial taxonomy at Species level of data-set TREATMENTS D07. [file 12864_2022_8638_MOESM13_ESM.tiff]

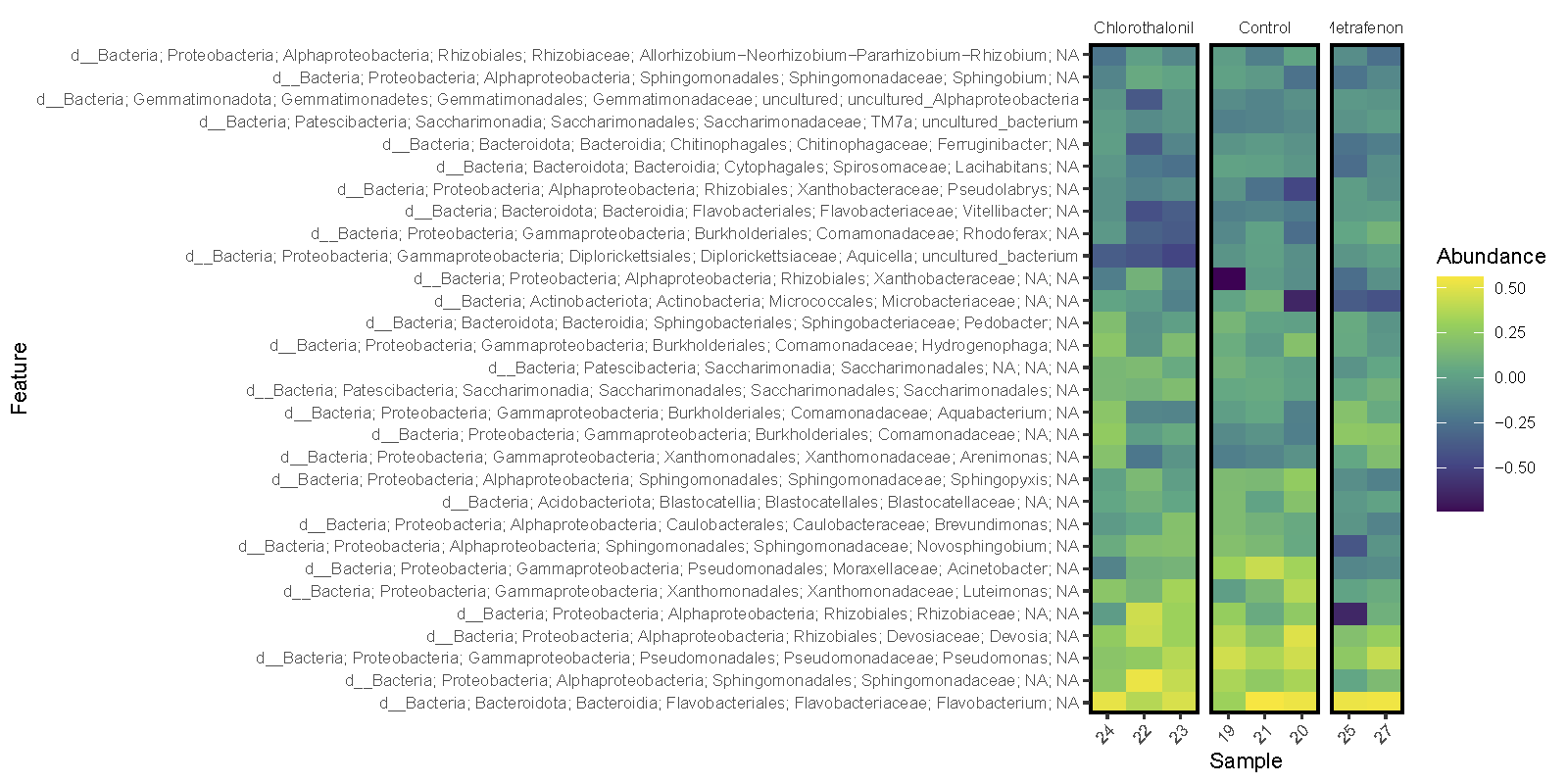

Supplement: Supplementary file 14 — Additional file 14: Supplementary Figure 11. Heatmap with the bacterial taxonomy at Species level of data-set TREATMENTS D16. [file 12864_2022_8638_MOESM14_ESM.tiff]

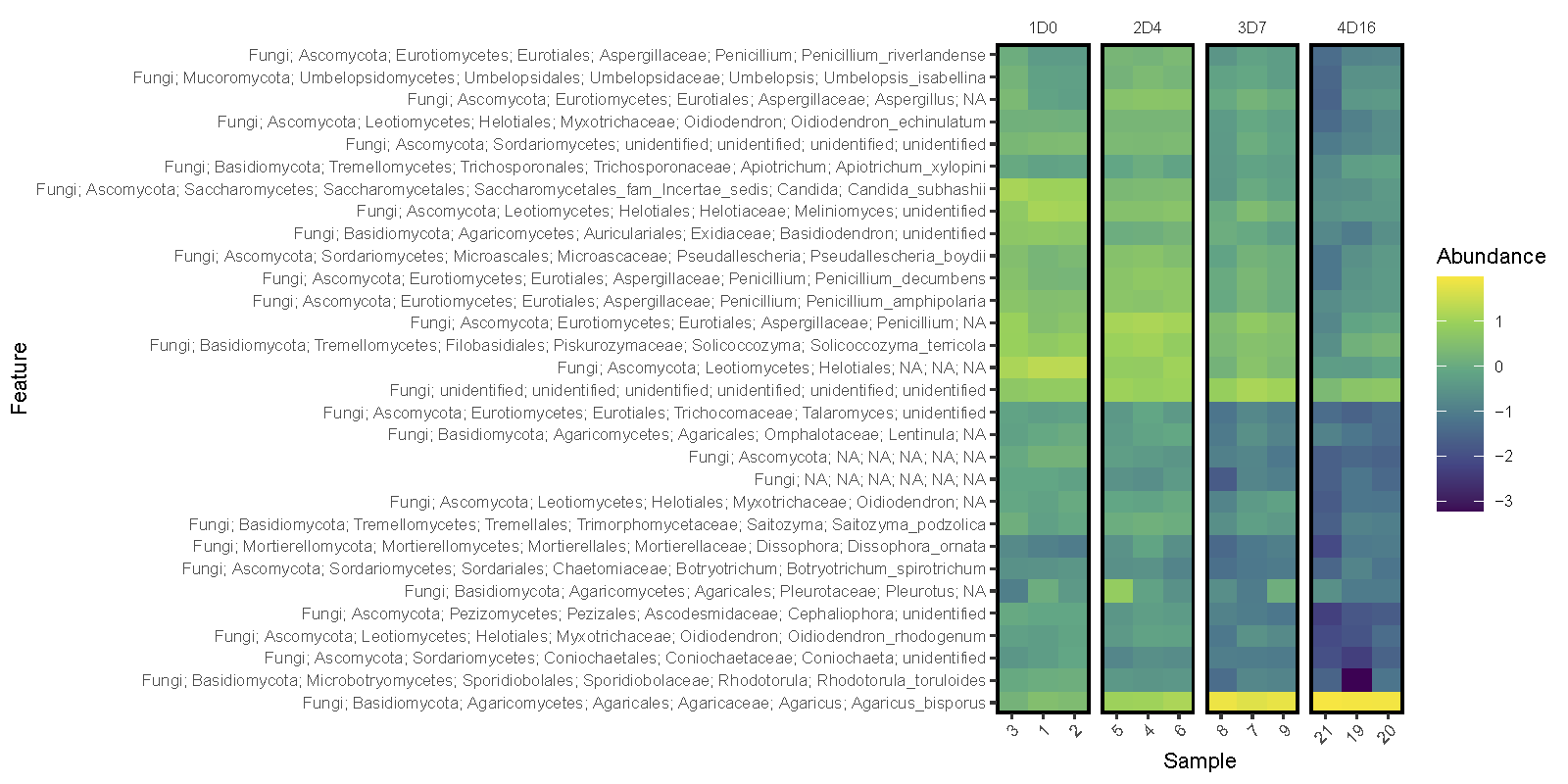

Supplement: Supplementary file 15 — Additional file 15: Supplementary Figure 12. Heatmap with the fungal taxonomy at Species level of data-set DAYS. [file 12864_2022_8638_MOESM15_ESM.tiff]

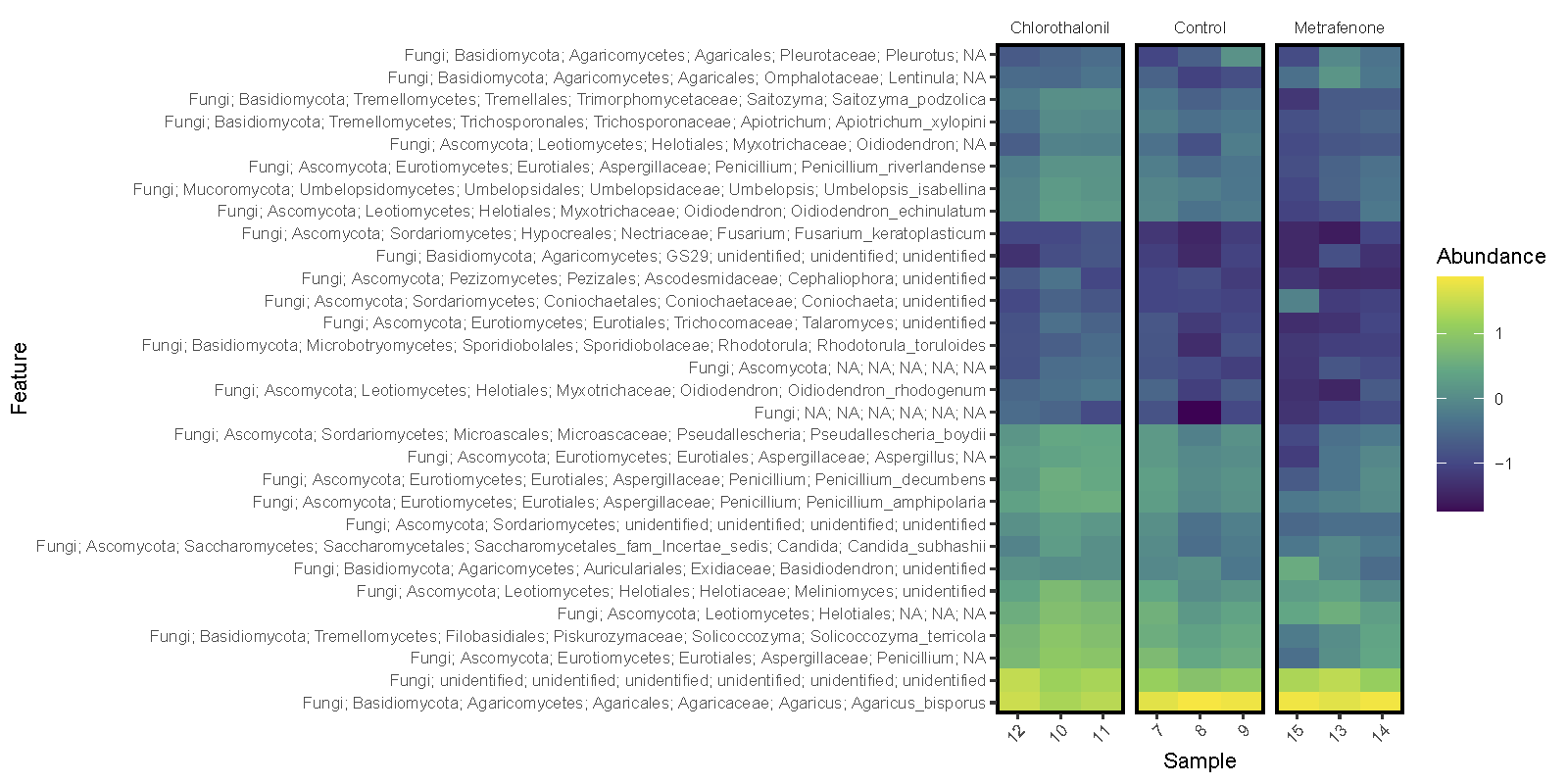

Supplement: Supplementary file 16 — Additional file 16: Supplementary Figure 13. Heatmap with the fungal taxonomy at Species level of data-set TREATMENTS D07. [file 12864_2022_8638_MOESM16_ESM.tiff]

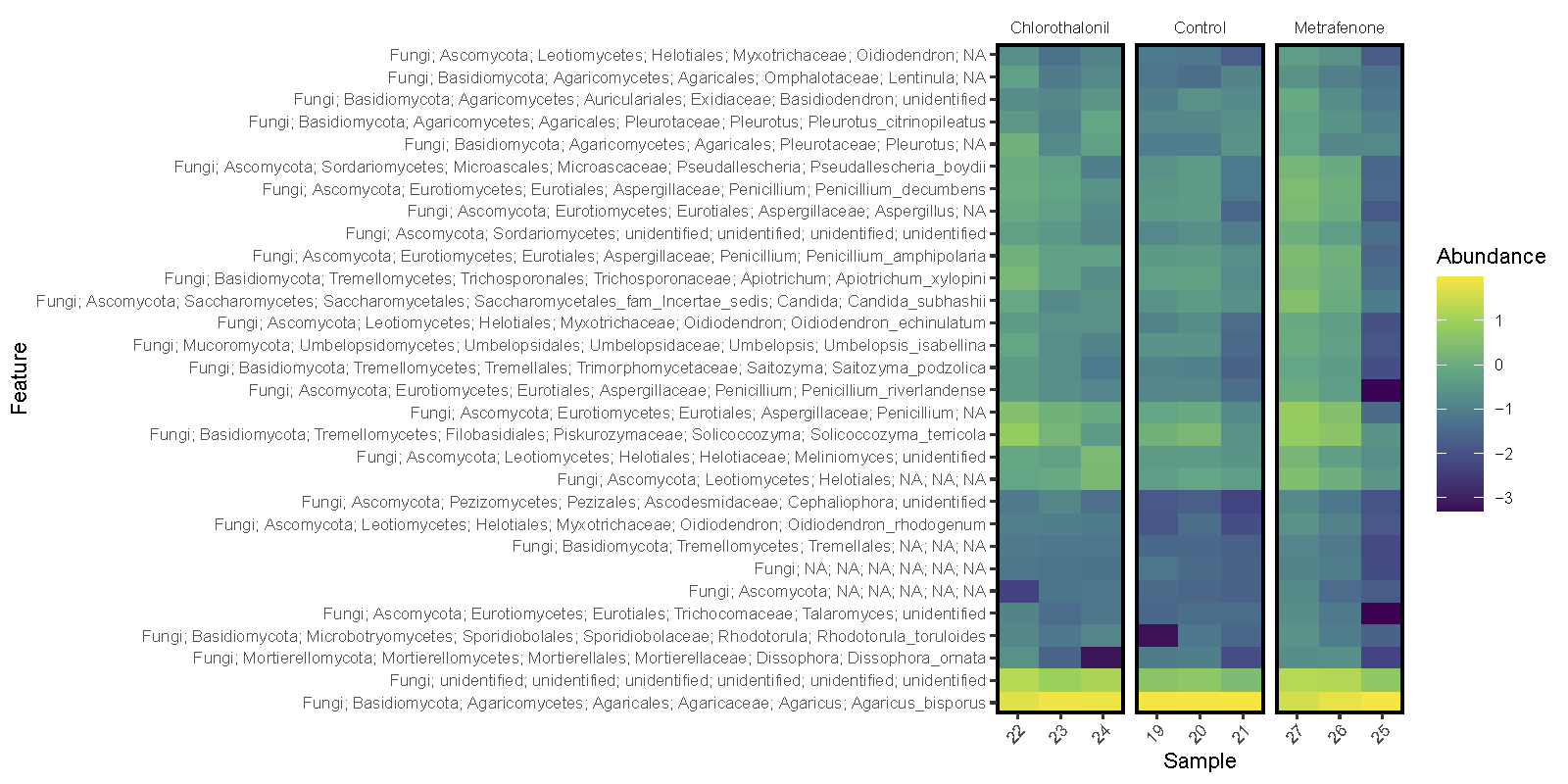

Supplement: Supplementary file 17 — Additional file 17: Supplementary Figure 14. Heatmap with the fungal taxonomy at Species level of data-set TREATMENTS D16. [file 12864_2022_8638_MOESM17_ESM.tiff]
